# Co-deleting *Pten* with *Rb* in retinal progenitor cells in mice results in fully penetrant bilateral retinoblastomas

**DOI:** 10.1186/s12943-015-0360-y

**Published:** 2015-04-24

**Authors:** Chencheng Xie, Huarui Lu, Alice Nomura, Eric Allan Hanse, Colleen Lynn Forster, Josh Berken Parker, Michael Andrew Linden, Chris Karasch, Timothy Curtis Hallstrom

**Affiliations:** Department of Pediatrics, University of Minnesota, Minneapolis, MN 55455 USA; Department of Surgery, University of Minnesota, Minneapolis, MN 55455 USA; Department of Laboratory Medicine and Pathology, University of Minnesota, Minneapolis, MN 55455 USA; BioNet, Academic Health Center, University of Minnesota, Minneapolis, MN 55455 USA; Department of Veterinary Population Medicine, University of Minnesota, St Paul, MN 55108 USA

**Keywords:** RB, E2F1, Retinoblastoma, PTEN, Apoptosis

## Abstract

**Background:**

*Rb1* is the most frequently mutated gene in the pediatric cancer retinoblastoma, and its loss causes E2F transcription factors to induce proliferation related genes. However, high E2F levels following pRB loss also induce apoptosis-promoting genes as a safeguard mechanism to suppress emergent tumors. Although p53 accumulation and apoptosis induction is believed to be a primary mechanism to eliminate cells with excess E2F activity, p53 deletion doesn’t suppress RB/E2F induced apoptosis *in vivo* in the retina. This prompted us to test the PTEN/PI3K/AKT signaling pathway on RB/E2F apoptosis suppression *in vivo*, to ascertain if the PI3K pathway may provide a potential avenue for retinoblastoma therapy.

**Methods:**

We developed a mouse model in which *Rb1* and *Pten* were conditionally deleted from retinal progenitor cells using *Chx10-Cre*, whereas *Rbl1* (p107) was constitutively deleted. Pathway components were also tested individually by *in vivo* electroporation into newborn retinas for an effect on apoptosis and tumor initiation. Mouse retinal tissues were analyzed by immunohistochemistry (IHC) for proliferation, apoptosis, and pathway activation. ShRNAs were used in vitro to assess effects on apoptosis and gene expression.

**Results:**

Co-deleting *Pten* with *Rb1* and *Rbl1* in mouse retinal progenitor cells (RPCs) causes fully penetrant bilateral retinoblastomas by 30 days and strongly suppresses Rb/E2F-induced apoptosis. *In vivo* electroporation of constitutively active (ca)-*Pik3ca*, ca-*Akt*, or dominant-negative (dn)-*Foxo1* into apoptosis prone newborn murine retina with deleted Rb/p107 eliminate Rb/E2F induced apoptosis and induce retinoblastoma emergence. Retinal deletion of Pten activates p-AKT and p-FOXO1 signaling in incipient retinoblastoma. An unbiased shRNA screen focusing on Akt phosphorylation targets identified FOXOs as critical mediators of Rb/E2F induced apoptosis and expression of Bim and p73 pro-apoptotic genes.

**Conclusions:**

These data indicate that we defined a key molecular trigger involving E2F/FOXO functioning to control retinal progenitor cell homeostasis and retinoblastoma tumor initiation. We anticipate that our findings could provide contextual understanding of the proliferation of other progenitor cells, considering the high frequency of co-altered signaling from RB/E2F and PTEN/PI3K/AKT pathways in a wide variety of normal and malignant settings.

**Electronic supplementary material:**

The online version of this article (doi:10.1186/s12943-015-0360-y) contains supplementary material, which is available to authorized users.

## Background

Retinoblastoma is a rare pediatric cancer of the retina that is fatal if left untreated. Although success rates for retinoblastoma treatment are high (>90%) in the U.S., treatment often involves removal of one or both eyes and loss of vision. Also, many patients in developing countries refuse eye removal and die from tumor metastasis. Continued improvements in therapy are needed to reduce the need for this extreme treatment. This has prompted investigators to identify genetic pathways that control retinal development, and investigate how these pathways may provide molecular targets that contribute to retinoblastoma development so that they may be targeted therapeutically with chemical inhibitors in conjunction with standard chemotherapeutic regimens.

Retinal development demands that specific cell types are generated from RPCs in the proper numbers and positioned in the correct location [1]. Mature vertebrate retinae are comprised of seven cell types stratified into distinct layers: the outer nuclear layer (ONL), outer plexiform layer (OPL), inner nuclear layer (INL), inner plexiform layer (IPL), and ganglion cell layer (GCL). Co-deletion of the pRB and p107 pocket proteins increases the number of RPCs still present at birth and predisposes to a tumor phenotype, indicating a role for these proteins in retinal terminal differentiation [2-4].

pRB functions widely in human tumor suppression and regulates proliferation by binding to and inhibiting the E2F family of transcription factors. However, E2F1 also induces an apoptotic gene expression program, which has been postulated to suppress tumor initiation by eliminating cells that have acquired a single oncogenic mutation in the RB pathway [5]. Surprisingly, deep sequencing of human retinoblastoma tumors has uncovered an extremely low mutation rate, with only the *RB1* gene itself emerging as a highly mutated gene in this cancer [6]. Thus, it is unclear exactly how apoptosis is suppressed in retinal tissue upon RB inactivation during normal development or retinoblastoma tumor initiation.

E2F1 is required for pro-apoptotic signaling following pocket protein deletion in the retina [7]. It is widely believed that the p53 tumor suppressor protein is the primary apoptotic effector of deregulated E2F activity. E2Fs can directly induce p14^ARF^ expression, which binds and inhibits MDM2, an E3 ubiquitin-ligase for p53 [8]. A number of studies have pointed towards an indirect loss of the p53 pathway in mouse and human retinoblastomas, through alterations in levels or function of p19^ARF^, MDM2 or MDMX [9-12]. However, RB/E2F induced cell death is not attenuated in retinal cells deficient in *Rb1/p107/Trp53* or *CDKN2B (p19*^*ARF*^*)* [2,9,13], although MDMX overexpression can block cell death [11]. Likewise, high MDM2 blocks cell death in Rb-deficient cone precursor cells [14].

This prompted us to test the PTEN/PI3K/AKT signaling pathway on RB/E2F apoptosis suppression *in vivo*, which can block RB/E2F-induced cell death in tissue culture models, to ascertain if the PI3K pathway may provide a potential avenue for retinoblastoma treatment [15,16]. The *PTEN* tumor suppressor gene encodes a lipid phosphatase that antagonizes phosphatidylinositol-3 kinases (PI3K) by dephosphorylating phosphatidylinositol 3,4,5-triphosphate, and both genes are frequently lost in many human cancers [17,18]. PTEN loss or PI3K activation leads to activation of AKT, a serine/threonine kinase that directly phosphorylates a wide variety of targets to control survival, protein synthesis and glucose metabolism [19]. Several genetic alterations in retinoblastoma implicate PTEN/PI3K/AKT pathway activation. First, activating mutations in PIK3CA have been detected in human retinoblastoma [20]. Second, the *SYK* proto-oncogene kinase, a strong activator of PI3K/AKT signaling in other cancers such as diffuse large B-cell lymphomas, is epigenetically modified and upregulated in some retinoblastomas to suppress apoptosis [6,21]. Third, the *MIRC1* (miR-17-92) microRNA cluster, which can activate AKT in a variety of contexts, is amplified and linked to cell death suppression in human retinoblastoma, and its overexpression in retinal cells with *Rb1* and *p107* deletion promotes rapid retinoblastoma [22-24].

To better understand how RB/E2F and PTEN/PI3K/AKT pathways control RPC homeostasis and apoptosis *in vivo*, we co-deleted *Rb1*, *Rbl1* (p107) and *Pten*, in mouse RPCs. Their co-deletion suppressed RB/E2F induced cell death, unlike p53 deletion, and promoted rapid retinoblastoma emergence. We found that the PI3KCA/AKT/FOXO1 signaling pathway mimics Pten deletion *in vivo* in the retina, converging with the RB/E2F pathway to control apoptosis and retinoblastoma tumor formation. FOXOs are a family of transcription factors (*FoxO1*, *FoxO3*, *FoxO4*, and *FoxO6*) present in all eukaryotes that function in cell death, cell-cycle arrest, DNA repair, cell differentiation, glucose metabolism, and protection from oxidative stress. Their phosphorylation by AKT causes their inactivation through loss of DNA binding activity or cytoplasmic relocalization [25]. It has recently been shown that the E2F1 and FoxO1 transcription factors physically associate to control expression of the pro-apoptotic *Apaf1* gene *in vitro*, but the effects of this complex in suppressing tumor onset *in vivo* is completely unknown [26]. Our study reveals that the E2F1/FOXO1 complex suppresses retinoblastoma emergence by inducing cell death in the retina, in part through their combined transcriptional regulation of the retinal pro-apoptotic gene *Bim*. Because E2F1 and FOXO1 are not normally simultaneously functional in the nucleus, they require certain oncogenic stresses, such as loss of RB function, to trigger an apoptotic response and suppress tumor emergence. As such, E2F1/FOXO1 apoptotic and tumor suppressive functions are disabled during normal growth, and in emerging tumors, by the coordinated signaling of the PI3KCA and AKT, which directly disables FOXO1 function.

## Results

### Co-deleting *Pten* with *Rb1* in retinal progenitor cells in mice induces bilateral retinoblastoma

**Figure 1:**
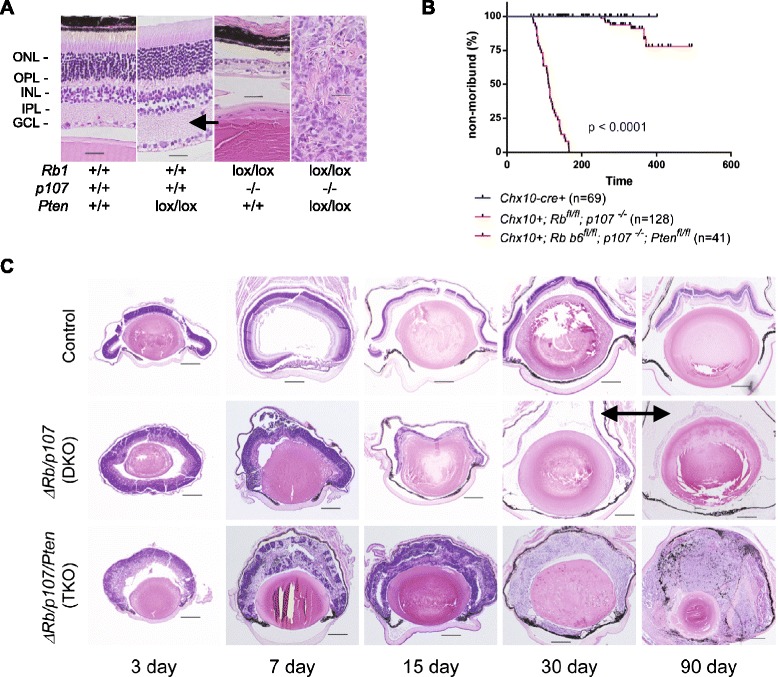
PTEN suppresses cell death and tumor emergence caused by RB/p107 loss in the retina. All mice are positive for *CHX10-cre*. **A**. H&E stained sections (representative of n = 3) from 8-month-old mice (except TKO; 5 month old) of the stated genotypes, Scale bar, 20 μm. **B**. Kaplan-Meier analysis for the % non-moribund status of the indicated mice. *P* values were determined by log rank test comparing a genotype with that of the control mice. **C**. H&E-stained sections from mouse eyes of the stated genotypes at 3, 7, 15, 30 and 90 days of age. Scale bar, 200 μm. See also Additional file 1: Figure S1.

To study the functional connection between the RB and PTEN pathways *in vivo* during retinal development, floxed Pten mice were mated with *Rb*^*lox/lox*^*, p107*^*−/−*^ and *Chx10-Cre*, (the latter expresses Cre recombinase in small clusters of RPCs beginning around embryonic day 10.5) [27]. Retina from control, *Chx10-Cre; Pten*^*lox/lox*^, *Chx10-Cre; Rb*^*lox/lox*^*; p107*^*−/−*^, hereafter referred to as double knockout (DKO), and *Chx10-Cre; Rb*^*lox/lox*^*; p107*^*−/−*^*; Pten*^*lox/lox*^, referred to as triple knockout (TKO) were harvested at 8 months (TKO were harvested at 5 months because none of them lived to 8 months) for hematoxylin and eosin (H&E) analysis (Figure 1A). We observed slightly increased retinal thickness in the IPL of *Pten*-deleted retina at 8 months, compared with control mice (see arrow), but we did not observe tumor development or any other notable disorganization of retinal stratification (n = 3). Consistent with previous observations, co-deleting *Rb1* and *p107* left only a hypocellular intrinsically death-resistant layer of cells [3,13,28]. In striking contrast, co-deleting *Pten* with *Rb1* and *p107* in RPCs induced rapid bilateral retinoblastoma formation. TKO mice exhibited dramatically increased moribundity compared with control or DKO mice, requiring euthanasia due to excessive tumor burden (Figure 1B). The rapid onset of these tumors prompted us to examine their formation over a 90-day time course (Figure 1C and Additional file 1: Figure S1). Control retinas appeared normal throughout the time course. *Rb1* and *p107* deletion progressively damaged the retina over 30 days, leaving just the thin band of death-resistant cells also observed at 8 months (see arrow). By contrast, *Pten* co-deletion caused fully penetrant bilateral retinoblastoma by 30 days and filled the entire ocular cavity by 90 days.

We next defined the cell types that abnormally proliferate in retinoblastomas of *Chx10-Cre; Rb*^*lox/lox*^*; p107*^*−/−*^*; Pten*^*lox/lox*^ mice to compare them with those from previous mouse retinoblastoma models [3,29]. Retinoblastomas arising in *Chx10-Cre; Rb*^*lox/-*^*; p107*^*−/−*^*; p53*^*lox/-*^ mice express PAX6 and Syntaxin which are markers expressed in progenitor & amacrine cells, and PKCα which is expressed in differentiated bipolar cells [29]. Retinoblastoma tumor cells from α-Cre; *Rb*^*lox/lox*^*; p107*^*−/−*^ mice express Syntaxin (progenitor/amacrine cells), Calbindin (horizontal cells) and CRALBP (Müller glia) cellular markers. Eyes from control mice or from 30 day old *Chx10-Cre; Rb*^*lox/lox*^*; p107*^*−/−*^*; Pten*^*lox/lox*^ mice were analyzed by IHC with antibodies that partially recognize specific retinal cell types (Additional file 1: Figure S2). Most of the tumor cells expressed Syntaxin. Some groups of cells within a tumor expressed Calbindin whereas other groups did not, indicating that these tumor cells were not all identical. Many of the tumor cells expressed Brn3b protein which is present in ganglion cells. Many also weakly expressed CHX10, a protein present both in differentiated bipolar cells and retinal progenitor cells, but they also expressed PKCα (differentiated bipolar cells). The tumors lacked cells that expressed markers associated with rod cells (Rho4D2), cone cells (Cone Arrestin), and Müller glia (CRALBP). Thus, tumors arising in our *Chx10-Cre; Rb*^*lox/lox*^*; p107*^*−/−*^*; Pten*^*lox/lox*^ mice are similar to previous mouse retinoblastomas in that they also predominantly reflect amacrine and horizontal cell types but they also differ in that they contain ganglion cells but few Müller glia cells.

### Regulation of RB/E2F-induced apoptosis and tumor onset by PIK3CA, AKT, and FOXO1 *in vivo*

**Figure 2:**
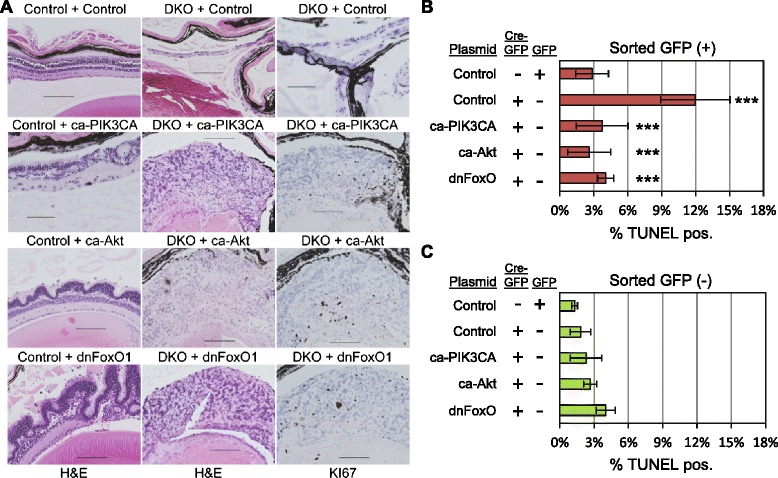
PIK3CA, AKT, and FOXO control RB/E2F apoptosis and tumor onset *in vivo*. **A**. Newborn *CHX10-Cre* (+) and *CHX10-cre* (+); *Rb1* ^*fl/fl*^ *; p107* ^*−/−*^ retina were electroporated *in vivo* with CAG-luciferase and control, constitutively active (ca) *PIK3CA*, ca-*AKT* plasmids, or dominant-negative (dn) *Foxo1*. Eyes were harvested at 60 days for H&E and KI-67 staining. **B**. Newborn *CHX10-Cre* (−); *Rb* ^*fl/fl*^ *;p107* ^*−/−*^ mouse retinas were transfected with GFP or Cre:GFP and either control plasmid, or plasmids that express ca-*PIK3CA*, ca-*AKT*, or *dnFoxo1*. Retinas were harvested 2 days post-electroporation, trypsinized into single cells, and sorted into GFP (+) and (−) cells by fluorescence-activated cell sorting (FACS). Individual GFP (+) retinal cells were quantified for TUNEL in triplicate at 48 hours. **C**. Individual GFP (−) retinal cells (counterparts from 4B) were quantified for TUNEL in triplicate at 48 hours. ***, p < 0.001. Scale bar, 100 μm.

We used *in vivo* electroporation in the mouse retina to ascertain if PIK3CA, AKT, and FOXO1 could substitute for PTEN loss in the control of RB/E2F-induced retinal apoptosis and tumor emergence. This technique has been used to study the role of transcription factors and other signaling genes in both normal retinal development and retinoblastoma induction [30,31]. We electroporated newborn control or DKO retina with control plasmids or plasmids expressing *ca-PIK3CA* (*E545K*), *ca-AKT* (myristoylated), or dominant negative (dn) *Foxo1*. DKO mice retain numerous proliferative RPCs at birth due to early RB deletion [3,4]. DnFOXO1 was used to block activity of all FOXOs and avoid a compensatory response. Control mice transfected with control plasmid developed retina appropriately by 2 months, whereas expression of ca-AKT or dnFOXO1 into Chx10^+^ mice caused a striking alteration in ONL architecture and development of hyper-cellularity at 2 months (Figure 2A). Control plasmid expression in DKO retina does not rescue the apoptotic cell loss or induce retinal tumors by 60 days. We did not detect tumor formation in control mice transfected with either control or dnFOXO1 plasmids. Transfection of ca-PIK3CA, ca-AKT, or dnFOXO1 into newborn DKO mouse retinas each led to striking tumor formation, with many Ki-67-positive cells, at 60 days of age (n = 5).

We next determined if ca-PI3KCA, ca-AKT, or dnFOXO can suppress apoptosis caused by RB/p107 deficiency in the retina. Newborn *(Chx10-) Rb*^*lox/lox*^*; p107*^*−/−*^ mouse retina were co-electroporated *in vivo* with plasmids expressing either GFP or CRE:GFP fusion with either a control, ca-PIK3CA, ca-AKT, or dnFOXO1 plasmid. Because these mice were *Chx10-*, the only source of CRE and GFP were added exogenously. Retinal cells were harvested and dissociated at 48 hours, FACS sorted into GFP (+) and GFP (−) populations which were then quantified for TUNEL. Around 3% of the GFP (+) transfected control cells were apoptotic (Figure 2B). The number of apoptotic cells increased to 12% following transfection with the CRE:GFP fusion plasmid and control in the GFP-isolated cells. Co-introducing ca-PIK3CA, ca-AKT, or dnFOXO1 with CRE:GFP each significantly reduced apoptotic levels to that caused by GFP + control plasmids alone. GFP (−) cells were also assayed in each experiment and were always around the 3% baseline, indicating that GFP transfection alone does not affect apoptotic levels in this assay (Figure 2C).

We determined if these tumors also expressed the same cellular marker seen in the *Chx10-Cre; Rb*^*lox/lox*^*; p107*^*−/−*^*; Pten*^*lox/lox*^ tumors or if they were different. Sections from the ca-PIK3CA, ca-Akt, and dnFOXO1 induced tumors were processed for IHC using the anti-Syntaxin and anti-Calbindin antibodies (Additional file 1: Figure S3). We observed that each of these tumors expressed Calbindin and Syntaxin, suggesting that the tumor cell composition was very similar to the tumors derived from *Chx10-Cre; Rb*^*lox/lox*^*; p107*^*−/−*^*; Pten*^*lox/lox*^ mice.

### p-Akt and p-FoxO1 signaling feature prominently in normal retina and ΔPten driven retinoblastomas

**Figure 3:**
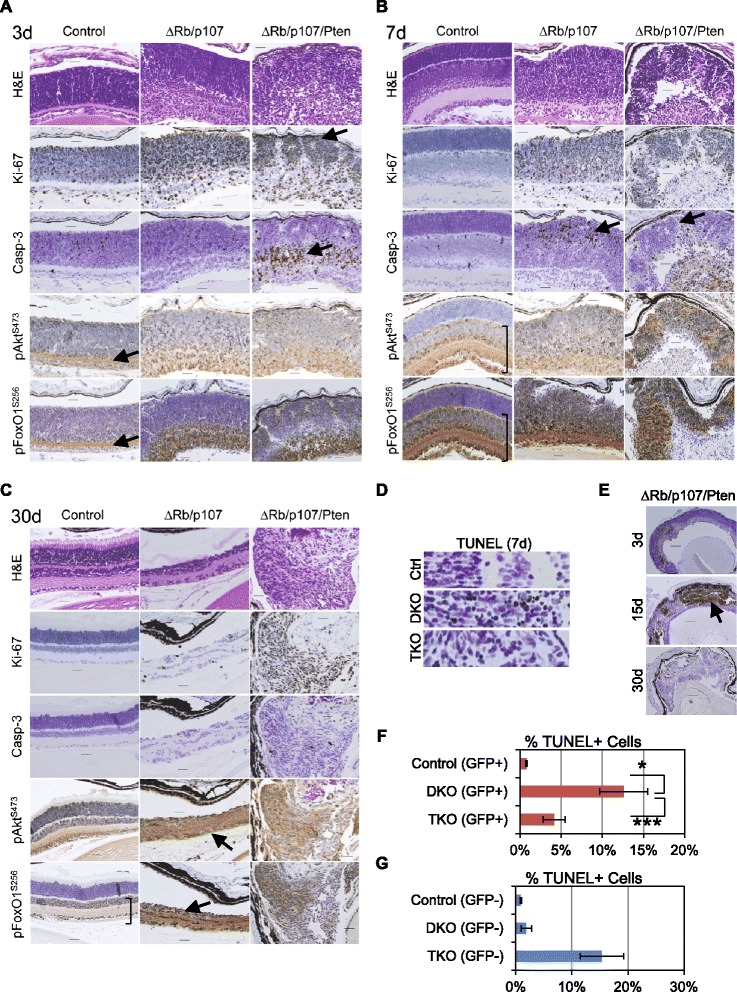
p-AKT and p-FOXO1 signaling feature prominently in normal retina and ΔPTEN-driven retinoblastomas. **A**-**C**. Mouse eyes from control, *Chx10-cre; Rb1* ^*fl/fl*^ *; p107* ^*−/−*^, or *Chx10-cre; Rb1* ^*fl/fl*^ *; p107* ^*−/−*^ *; Pten* ^*fl/fl*^ were harvested at 3 **(A)**, 7 **(B)**, and 30 **(C)** days after birth, fixed, embedded in paraffin wax, and sectioned for H&E staining or IHC analysis using specific antibodies for Ki-67, caspase-3, p-AKT (Ser473), or p-FOXO1 (Ser256). Scale bar, 20 μm. **D**. TUNEL staining in the retina of the listed mouse genotypes. **E**. Mouse eyes of *Chx10-cre; Rb1* ^*fl/fl*^ *; p107* ^*−/−*^ *; Pten* ^*fl/fl*^ (TKO) were harvested at 3, 15, and 30 days after birth, fixed, embedded in paraffin wax, and sectioned for caspase-3 IHC analysis. Scale bar, 100 μm. **F**. Quantification of TUNEL-positive Cre:GFP (+) sorted cells from these mice (n = 3). **G**. Quantification of TUNEL positive Cre:GFP (−) sorted cells from these mice (n = 3). Data are mean from samples analyzed in triplicate, and error bars represent standard deviation.

We used IHC staining for Ki-67, Caspase-3, terminal dUTP nick end labeling (TUNEL), p-AKT and p-FOXO1 to clarify how loss of PTEN and RB/p107 in the retina controls apoptosis and induces retinoblastoma. Ki-67 staining revealed comparable proliferation between control and DKO retina in all retinal layers at 3 days. Closer examination of 3-day-old TKO retina revealed numerous highly proliferative rosette-like structures (see arrow) (n = 3) (Figure 3A). Control retinas ceased proliferating at 7 days (Figure 3B), whereas Ki-67 staining was still present in all regions of the 7-day-old DKO retina, and was particularly robust in the TKO mice. By 30 days, however, the majority of DKO retinal tissue had been lost and few cells were proliferating, but the TKO mice had developed bilateral tumors (Figure 3C).

We determined if *Pten* deletion suppressed RB/E2F-induced apoptosis in *Rb1*/*p107* co-deleted RPCs using IHC. Each strain displayed considerable retinal apoptosis by caspase-3 IHC at 3 days (Figure 3A). Minimal caspase-3 staining was evident within the INL of 7-day-old control retina. Similar cell death was also seen in the DKO retina; however, high levels of apoptosis were seen predominantly in the ONL, the section that was lost by 30 days (see arrow). The rosette-like structures in TKO retina, however, were largely devoid of caspase-3 staining at 7 days (see arrow). At 30 days, very little apoptosis was observed in the highly proliferative tumors seen in TKO retina. TUNEL analysis confirmed that *Rb1/p107* deficiency induced massive retinal cell death at 7 days (~25% cells) which was countered by *Pten* co-deletion (Figure 3D). Caspase-3 IHC unexpectedly revealed sizable regions of apoptosis in TKO retina (Figure 3A,B,E).

The *Chx10-Cre* strain fused GFP with CRE recombinase, so we isolated CRE:GFP (+) and (−) cells from control, DKO, and TKO retinas to determine if these apoptotic cells were CRE:GFP (+) or (−). The number of CRE:GFP (+) TUNEL-positive cells increased from 2% in control retina to 13% in DKO retina and was significantly reduced to near control levels upon Pten co-deletion, indicating that Pten deletion mediates cell-intrinsic suppression of RB/E2F apoptosis (Figure 3F). CRE-GFP (−) cells from control and DKO retina remained at baseline levels for apoptosis. By contrast, CRE-GFP (−) cells isolated from TKO retina stained TUNEL-positive (16%) (Figure 3G). It is unclear what caused excess cell death in the CRE-GFP (−) cells but it may be due to reduced access to trophic factors or stress from improper cellular positioning.

We assessed the status of phospho-AKT (pAKT^Ser473^) and phospho-FOXO1 (pFOXO1^Ser256^) *in vivo* in control, DKO and TKO retina. pAKT^Ser473^ and pFOXO1^Ser256^ staining were detected in the IPL (see arrow) and throughout the neuroblastic layer of day 3 control retina (Figure 3A). The INL, IPL and GCL of control retina at days 7 and 30 displayed robust pAKT^Ser473^ and pFOXO1^Ser256^ staining, indicating that this signaling is a normal occurrence during retinal development (see brackets). In DKO retina, pAKT^Ser473^ and pFOXO1^Ser256^ signaling was strong in these same layers. By 30 days, the DKO retina was reduced to a p-AKT- and p-FOXO1-positive, apoptosis-resistant layer (see arrows). Our findings suggest that these cells might evade apoptosis in part because of increased p-AKT and p-FOXO activity. The highly proliferating regions of TKO retina displayed pAKT^Ser473^ and pFOXO1^Ser256^ beginning at 3 days, which was strong at 7 days, and robust in tumors at 30 days. pFOXO1^Ser256^ staining was observed in both the nucleus and cytoplasm of most of the affected cells, indicating that nuclear exclusion of FOXO1 is not an obligatory consequence of AKT phosphorylation in the retina. These data indicate that p-AKT and p-FOXO1 signaling is a normal event during retinal development.

### RB/E2F1-induced apoptosis requires FOXO1

**Figure 4:**
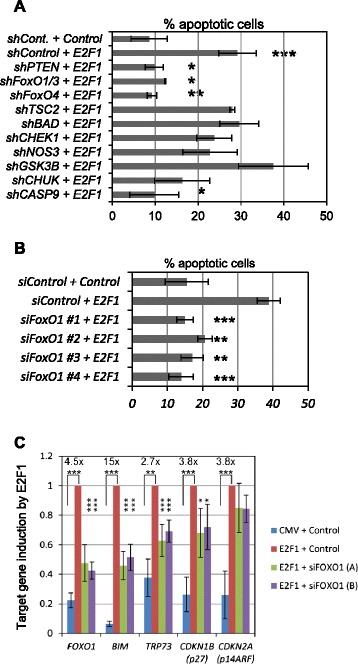
RB/E2F1-induced apoptosis requires FOXO1. **A**. REF52 cells were stably integrated with shRNAs specific for AKT phosphorylation targets, infected with control or E2F1-expressing adenovirus, and measured for active caspase-3 levels (n = 3). **B**. U2OS cells were transfected with control or four individual *FOXO1* siRNAs (n = 3), the deprived of serum, infected with adeno-E2F1, and measured for cleaved caspase-3 levels. **C**. Y79 cells transfected with control or FoxO1 siRNAs were infected with control or AdE2F1 and gene expression of *BIM*, *TRP73*, *CDKN1B,* (p27^KIP1^) and *CDKN2A* (p14^ARF^) was measured (n = 3). The fold induction by E2F1 is listed above each set. *, p < 0.05; **, p < 0.01; ***, p < 0.001. Error bars represent standard deviation from the mean.

We used an unbiased shRNA screen focusing on AKT phosphorylation targets to identify critical regulators of E2F1 transcriptional and apoptotic function. shRNA expressing Lentivirus were stably integrated into the genome of REF52 (immortalized rat embryo fibroblast) cells, which do not contain known mutations in the PTEN/PI3K/AKT pathway, and remain responsive to growth-factor stimulation and serum deprivation. Stable shRNA integrants were measured for target gene knockdown by quantitative PCR (qPCR) (Additional file 1: Figure S4A). Some targets (*Mdm2, Raf, Mtor, Cdkn1a*) failed to be knocked down and are not shown. E2F1 administration induced caspase-3 cleavage in 29% of the control cells compared with control adenovirus, which induced caspase-3 cleavage in less than 10% of the cells (Figure 4A). *Tsc2*, *Chek1*, *Nos3* and *Gsk3b* shRNAs effectively knocked down targets by 80% but had no effect on E2F1 mediated cell death in this assay. Pten knockdown was 85% effective and reduced E2F1-induced caspase-3 cleavage back to control levels. Knockdown of “conserved helix-loop-helix ubiquitous kinase” (*Chuk*), a regulator of the NF-κB transcription factor, and *Casp9*, which encodes the Caspase-9 initiator caspase, also inhibited E2F1-mediated cell death.

One of our shRNAs co-targeted both *FoxO1* and *FoxO3* for degradation (40% knockdown of *FoxO1* and 50% knockdown of *FoxO3*). This construct did not affect *FoxO4* levels. Although knockdown of either *FoxO1* or *FoxO3* was not robust using this construct, their combined deletion led to a striking reduction in E2F1-induced apoptosis, reducing caspase-3 cleavage from 29% in control, to 12% in cells treated with small interfering RNA (siRNA), namely, *siFoxO1/FoxO3*. An *shFoxO4* targeting construct, which did not affect *FoxO1* or *FoxO3* mRNA levels (levels not shown), reduced E2F1 apoptotic potential to 9%, comparable to the *shFoxO1/FoxO3* construct. We targeted endogenous *FOXO1* in human U2OS cells using four different siRNAs constructs (Dharmacon) (Figure 4B), because our previous experiments did not account for potential off-targeting effects, and in one case targeted more than one FOXO. Control or *siFOXO1* transfected U2OS cells were infected with control adenovirus (ad) or *ad-E2F1* in low-serum (0.25%) media and harvested for flow cytometry analysis of sub-G1 apoptotic DNA content, using propidium iodide. Each of the *siFOXO1* nucleotides, but not the control, effectively diminished *FoxO1* levels (55 − 75% knockdown, (Additional file 1: Figure S4b) and significantly prevented *ad-E2F1*-mediated apoptosis (Figure 4B). Additionally, E2F1 and FOXO1 proteins associate, and their co-expression induces more apoptosis than either alone, and this can be inhibited by growth factor addition (Additional file 1: Figure S5).

We evaluated whether E2F1 requires FOXO1 to induce pro-apoptotic target gene expression in Y79 human retinoblastoma cells transfected separately with control or two *FoxO1* siRNAs. *FOXO1*, a known E2F target gene, is induced 4.5-fold by E2F1 in Y79 cells, and the two siRNAs effectively depleted *FoxO1* (Figure 4C) [32]. Analysis of E2F1-dependent gene expression in control or *FoxO1*-depleted Y79 cells indicated a significant reduction in expression of the pro-apoptotic *BIM*, *TRP73*, but not *CDKN2A* (p14^ARF^) in *siFoxO1*-treated cells. E2F1-dependent induction of the cell-cycle regulator *CDKN1B* (p27^KIP1^) was also reduced by *shFoxO1*.

## Discussion

Loss of the RB pathway in retinoblastoma and other cancers strongly activates E2F transcription factor function, which coordinates a large-scale gene expression program for the purpose of DNA replication and mitosis, but also of apoptosis and senescence as a putative safeguard mechanism. E2F1 co-deletion in Δ*Rb1/p107*-deleted retina potently blocks apoptosis, but tumors fail to develop because proliferation is also disabled, indicating that E2F1 is required for both pro-apoptotic and pro-proliferatory signaling upon pocket protein deletion [7]. This work demonstrates that *Pten* deletion, activation of the PI3K/AKT pathway, or suppression of FOXO activity eliminates cell death caused by E2F1 *in vivo* in the retina and also induces rapid, bilateral retinoblastoma emergence.

**Figure 5:**
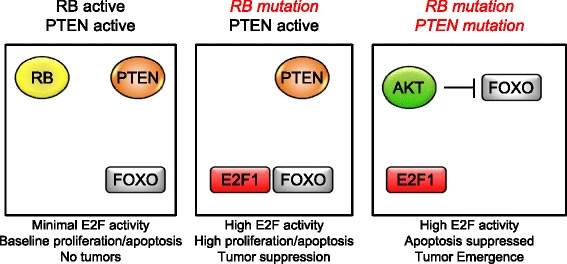
A model for the consequences of RB or PTEN deletion on E2F1/FOXO tumor suppressive apoptosis. RPCs with normal RB and PTEN protein levels do not proliferate due to minimal E2F activity. In the event of an RB mutation, E2Fs are deregulated and may complex with FOXO1 to induce apoptosis and suppress tumor emergence. Deleting PTEN activates p-AKT, causing p-FOXO1 inactivation and thereby disabling E2F-driven apoptosis, while allowing proliferation to occur. This model predicts that E2F and FOXO are simultaneously active in the nucleus only after certain oncogenic stresses, such as *Rb1* loss.

A model integrating these observations suggests that RPCs with normal RB and PTEN function do not proliferate owing to low E2F levels (Figure 5). Loss of RB/p107 activates E2F function to initiate proliferation; however, E2F1/FOXO apoptosis induction suppresses tumor initiation. Deleting PTEN activates p-AKT and enforces FOXO1 inactivation, thereby allowing E2Fs to continue their proliferative, but not apoptotic, target gene induction, which causes rapid bilateral retinoblastoma emergence. Because E2F1 and FOXOs are not normally simultaneously functional in the nucleus, they require certain oncogenic stresses, such as loss of RB function, to trigger an apoptotic response and suppress tumor emergence.

Other studies in a variety of species have also demonstrated an anti-apoptotic or pro-tumorigenic role for these pathways in the retina. Normal retinal development requires specific trophic factors, which induce differentiation concomitant with PI3K/AKT pathway activation. PI3K or AKT inactivation following growth-factor withdrawal induces expression of the FOXO target gene *Bim*, leading to ganglion cell death *in vitro* and *in vivo* [33]. Likewise, direct FOXO expression induces apoptosis in the retina of *Xenopus laevis* and *Drosophila melanogaster* [34,35]. PTEN overexpression in *D. melanogaster* eyes induces differentiating cells to undergo apoptosis, suggesting that AKT activation may be crucial for suppressing cell death during RPC exit from proliferation [36]. Transgenic activation of p65^PI3K^ in the mouse retina promotes retinal dysplasia and mediates cell survival, particularly in the neuroblastic layer [37]. This suggests that this abnormal proliferation could also pertain to the abnormal ONL morphology and increased cell number we see in our control mice transfected with ca-Akt and dnFOXO1 (Figure 2A). These pathways also link altered cell death to other retinal diseases. Insulin depletion *in vivo* in mice with underlying rod cell degeneration reduces PI3K activity and causes cell death in cone photoreceptors, leading to retinitis pigmentosa [38]. Diabetic retinopathy, which can lead to blindness, is an end stage effect of excess cone cell death and is also associated with decreased PI3K/AKT signaling [39]. Together, these findings indicate that excess PI3K/AKT pathway signaling in the retina can contribute to tumorigenesis, whereas its depletion potentially causes premature retinal degeneration.

RB and PTEN deletion appear to affect RPC homeostasis. Co-deletion of *Rb1* and *p107* in RPCs promotes RPC cell-cycle entry and self-renewal by delaying terminal differentiation, but also induces massive cell death [4,29]. *Pten* deletion in the retina leads to elevated proliferation of RPCs in day 0 pups, but by day 4 the proliferation decreases to a level below that of control retina [40]. This phenotype is reminiscent of *Pten* deletion in hematopoietic stem cells (HSCs), where it promotes proliferation, but not self-renewal, leading to stem cell exhaustion after several days [41]. The combined deletion of *Foxo1*, *Foxo3*, and *Foxo4* in HSCs comparably increases numbers of committed progenitors but decreases long-term HSC numbers, which exhibit reduced bone marrow repopulation in recipient mice [42,43]. Similar effects were observed in neural stem cells following combined *Foxo* deletion [44].

## Conclusions

Our studies have elucidated a molecular link between the RB and PTEN tumor suppression programs in RPC stem cell homeostasis. Loss of PTEN, or inactivation of FOXOs, in the retina do not induce tumorigenesis on their own. But when combined with the self-renewal promoting capacity of ΔRB/p107, the normally associated cell death is prevented and bilateral retinoblastomas quickly emerge. This model is potentially applicable to the wide variety of cancers that co-mutate RB and PI3K/PTEN (for example breast, ovarian, glioma, prostate, and lung), and it will be interesting to determine the extent to which other cells similarly activate PI3K and Akt to inactivate FoxOs and suppress cell death during RB/E2F mediated expansion.

## Methods

### Experimental animals

*CHX10-Cre* mice [27], Rb1^lox/lox^ mice [45], p107^−/−^ mice [46], and Pten^lox/lox^ mice [47] and related PCR genotyping protocols have been described. All mouse experiments were performed in accordance with University of Minnesota Institutional Animal Care and Use Committee procedures and guidelines. The University of Minnesota Comparative Pathology Shared Resource assisted with H&E staining, KI-67 & TUNEL IHC staining. Kaplan-Meier curves were calculated using GraphPad Prism software. P-values were determined by Student’s *t*-test.

### Cell culture and DNA plasmids

Cell culturing, adenovirus infections, caspase-3 and propidium iodide apoptosis assays were performed as described [48]. REF52 (rat embryo fibroblast) and U2OS (human osteosarcoma) cells were cultured in DMEM media containing 10% fetal calf serum. Y79 cells (ATCC) were cultured in RPMI1640 media containing 20% FBS. Individual Open Biosystems shRNAs were obtained from the University of Minnesota RNAi core facility. Three lentivirus per gene were co-transfected for packaging, and the final lentiviral mixture was used to infect target REF52 cells, followed by puromycin treatment to select for stable integrants. Dominant negative *Foxo1* (mouse, *Myc-FoxO1D256*, Addgene plasmid #12145), *Myr-Akt* (Addgene plasmid #9008), *CAG-Cre* (Addgene plasmid #13775), *CAG-Cre::GFP* (Addgene plasmid #13776), were obtained from Addgene. siRNAs for *FOXO1* were purchased as 4 individual nucleotides as SmartPools from Dharmacon and transfected into U2OS cells prior to apoptosis experiments.

### RNA isolation, real-time PCR & microarray analysis

RNA was prepared from cells for quantitative real-time PCR using RNeasy, QIAshredder, and QuantiTect SYBR Green RT-PCR kits from QIAGEN. Each RT-PCR experiment was performed in triplicate and normalized against expression of GAPDH expression levels and fold changes were calculated using ΔΔCT method. Primers are listed in Additional file 1: Table S1.

### Protein immunoblotting, co-immunoprecipitation, & immunohistochemistry

Immunoblotting and co-immunoprecipitations were performed as described [48]. Antiserum against FOXO (C29H4) and anti-Flag M2 (F3165) was purchased from Cell signaling Technology and Sigma for immunoblot analysis. E2F1 antibody (C20) was purchased from Sigma. Anti-HA antibody was purchased from Roche (3 F10). Anti-p53 (sc-6243, Santa Cruz), p-FOXO1 (9461S, Cell Signaling Technology), p-Akt (sc-7985-R, Santa Cruz), Capase-3 (Cell Signaling Technology, Cat# 9661), Ki-67 (SP6) (Biocare Medical, Cat# CRM325), and TUNEL (EMD Millipore, Cat# S7101) were used for IHC. IHC detection was through primary antibodies listed in Additional file 1: Table S2, with Vector biotinylated secondary (1:250), tertiary was streptavidin-horseradish peroxidase (Covance #SIG-32000) and chromagen 3,3′-diaminobenzidine substrate (Covance #SIG-31043).

### *In vivo* electroporation and mouse histology

Eyes were dissected from mice and fixed for approximately 18 hours in 10% neutral buffered formalin, then transferred to 70% ethanol and processed for histology. DNA was electroporated into the retina mice of the stated genotype using a CUY-21SC square wave electroporator following the Dyer lab protocol [11,30,31]. Experimental retina were harvested two days after transfection, trypsinized to single cells, and sorted into GFP^+^ and GFP^−^ populations. Recovered cells were placed on poly-lysine treated slides, fixed for 5 minutes in 4% paraformaldehyde, washed twice with 1× Dako wash buffer. Apoptotic cells were detected and quantified using a DeadEnd TUNEL kit (Promega #G7130) according to the manufacturer’s protocol followed by Cy3 (TSA fluorescence kit PerkinElmer #NEL704A) and Hoescht counterstain. Tumor induction studies were done by co-injecting DNA mixtures containing 2.5 μg CAG-luciferase plasmid and 2.5 μg experimental plasmids (*dnFoxo1*, ca-*Akt*, or ca-*PIK3CA*). Luciferase imaging was performed at 24 hours post-electroporation to leave untransfected pups out of the experimental pool. Successfully transfected pups were aged 2 months and eyes harvested, fixed, paraffin embedded, and sectioned for H&E staining.

## Additional file

Additional file 1: Figure S1. Related to Figure 1. Pten suppresses tumor emergence caused by Rb/p107 loss in the retina. H&E stained sections from *Chx10-Cre(+)* mouse eyes of the stated genotypes and timepoints. Scale bar, 50 μm. **Figure S2.** Related to Figures 1 & 3. ∆Rb/p107/Pten induced tumors express horizontal and amacrine cell markers. Retina from control mice or from *Chx10-Cre; Rblox/lox; p107-/-; Ptenlox/lox* were analyzed by IHC with the stated antibodies with specificity towards different retinal cell types (Calbindin: horizontal cell; Syntaxin: amacrine cell; Brn3b: ganglion cell; CRALBP: Müller glia; CHX10: bipolar cell, retinal progenitor cells; PKCα: bipolar cells; Rho4D2: rod cells; Cone arrestin: cone cells). Scale bar, 20μm. **Figure S3.** Related to Figure 2. Retinal tumors derived from *in vivo* electroporations using ca-PI3KCA, ca-Akt, and dnFOXO1 plasmids express horizontal (anti-calbindin) and amacrine (anti-syntaxin) cell markers. Scale bar, 20 μm. **Figure S4.** Related to Figure 4. Target gene knockdown levels by shRNA and siRNA. A. REF52 cells were stably integrated with shRNAs specific for the listed AKT phosphorylation targets and measured for target knockdown by qPCR. B. U2OS cells were transfected with control or four individual FoxO1 siRNAs and RNA isolated for qPCR detection of FoxO1 levels. **Figure S5.** Related to Figure 4. E2F1 and FOXO1 associate and promote cell death. A. Endogenous co-IPs. 293T human embryonic kidney and Y79 human retinoblastoma cells were lysed, and the extracts were immuno-precipitated with anti-E2F1 antisera and immunoblotted for FoxO1. B. Tagged co-IPs. 293T cells were transfected with HA-E2F1 and Flag-FoxO1, lysed, immunoprecipitated with anti-HA and blotted with anti-FLAG. C. E2F1 and FOXO1 were introduced into 0.25% (-) or 10% (+) serum treated U2OS cells and apoptosis measured. **Table S2.** shRNAs (A) and primers (B) used in this study. **Table S2.** Retinal Marker Antibodies used in this study.

## Abbreviations

ca: Constitutively active
dn: Dominant-negative
RPCs: Retinal progenitor cells
ONL: Outer nuclear layer
OPL: Outer plexiform layer
INL: Inner nuclear layer
IPL: Inner plexiform layer
GCL: Ganglion cell layer
DKO: Double knockout (*Chx10-Cr; Rb*^*lox/lox;*^*p107*^*−/−*^)
TKO: Triple knockout (*Chx10-Cre; Rb*^*lox/lox./*^*; p107*^*−/−*^*; Pten*^*lox/lox*^)
IHC: Immunohistochemistry
qPCR: Quantitative PCR
siRNA: Small interfering RNA
ad: Adenovirus
