# Supplementary material for: Co-deleting Pten with Rb in retinal progenitor cells in mice results in fully penetrant bilateral retinoblastomas
Source: Mol Cancer. 2015 Apr 24;14:93. doi: 10.1186/s12943-015-0360-y (PMC4411757; doi:10.1186/s12943-015-0360-y)
Supplement: Additional file 1: Figure S1. — Related to Figure 1. Pten suppresses tumor emergence caused by Rb/p107 loss in the retina. H&E stained sections from Chx10-Cre(+) mouse eyes of the stated genotypes and timepoints. Scale bar, 50 μm. Figure S2. Related to Figures 1 & 3. ∆Rb/p107/Pten induced tumors express horizontal and amacrine cell markers. Retina from control mice or from Chx10-Cre; Rblox/lox; p107-/-; Ptenlox/lox were analyzed by IHC with the stated antibodies with specificity towards different retinal cell types (Calbindin: horizontal cell; Syntaxin: amacrine cell; Brn3b: ganglion cell; CRALBP: Müller glia; CHX10: bipolar cell, retinal progenitor cells; PKCα: bipolar cells; Rho4D2: rod cells; Cone arrestin: cone cells). Scale bar, 20μm. Figure S3. Related to Figure 2. Retinal tumors derived from in vivo electroporations using ca-PI3KCA, ca-Akt, and dnFOXO1 plasmids express horizontal (anti-calbindin) and amacrine (anti-syntaxin) cell markers. Scale bar, 20 μm. Figure S4. Related to Figure 4. Target gene knockdown levels by shRNA and siRNA. A. REF52 cells were stably integrated with shRNAs specific for the listed AKT phosphorylation targets and measured for target knockdown by qPCR. B. U2OS cells were transfected with control or four individual FoxO1 siRNAs and RNA isolated for qPCR detection of FoxO1 levels. Figure S5. Related to Figure 4. E2F1 and FOXO1 associate and promote cell death. A. Endogenous co-IPs. 293T human embryonic kidney and Y79 human retinoblastoma cells were lysed, and the extracts were immuno-precipitated with anti-E2F1 antisera and immunoblotted for FoxO1. B. Tagged co-IPs. 293T cells were transfected with HA-E2F1 and Flag-FoxO1, lysed, immunoprecipitated with anti-HA and blotted with anti-FLAG. C. E2F1 and FOXO1 were introduced into 0.25% (-) or 10% (+) serum treated U2OS cells and apoptosis measured. Table S2. shRNAs (A) and primers (B) used in this study. Table S2. Retinal Marker Antibodies used in this study. [file 12943_2015_360_MOESM1_ESM.pdf]

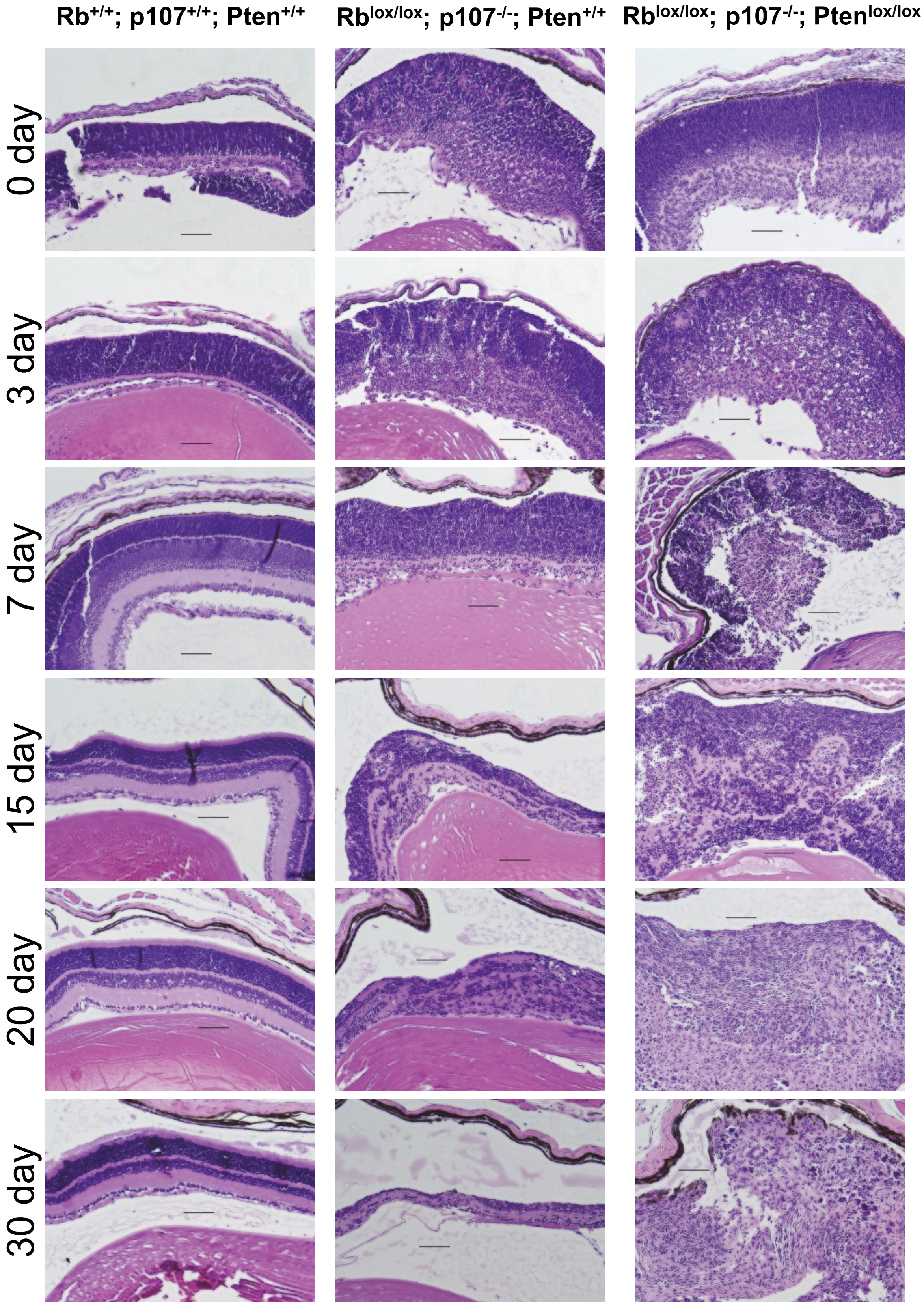

Figure S1, Related to Figure 1. Pten suppresses cell death and tumor emergence caused by Rb/p107 loss in the retina. H&E stained sections from mouse eyes of the stated genotypes at 0, 3, 7, 15, 20, and 30 days of age. All mice are positive for CHX10-cre. Scale bar, 50mm.

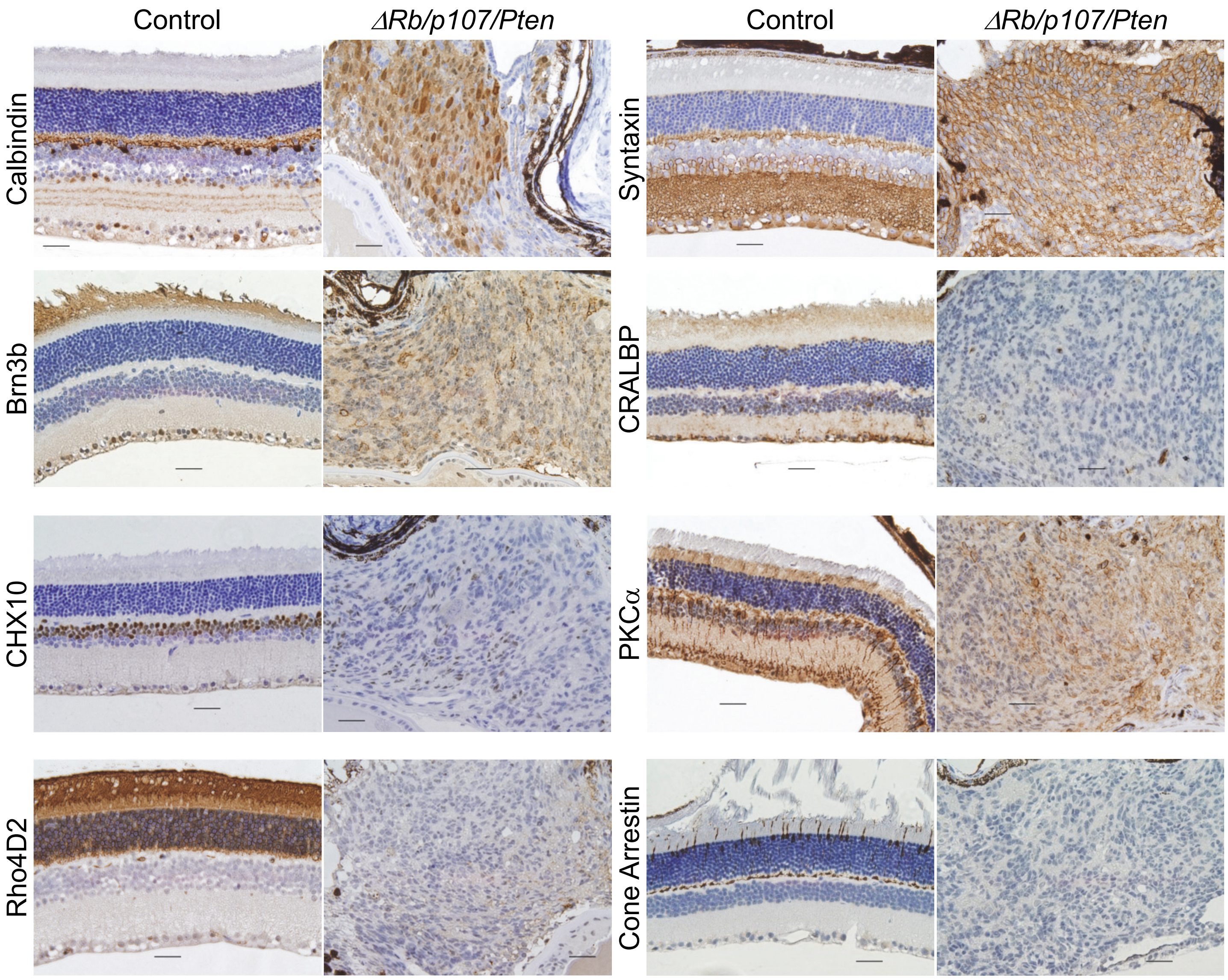

Figure S2, Related to Figures 1 & 3. ΔRb/p107/Pten induced tumors express horizontal and amacrine cell markers. Retina from control mice or from *Chx10-Cre; Rb*$^{lox/lox}$; *p107*$^{-/-}$; *Pten*$^{lox/lox}$ were analyzed by IHC with the stated antibodies with specificity towards different retinal cell types (Calbindin: horizontal cell; Syntaxin: amacrine cell; Brn3b: ganglion cell; CRALBP: Müller glia; CHX10: bipolar cell, retinal progenitor cells; PKCα: bipolar cells; Rho4D2: rod cells; Cone arrestin: cone cells). Scale bar, 20mm.

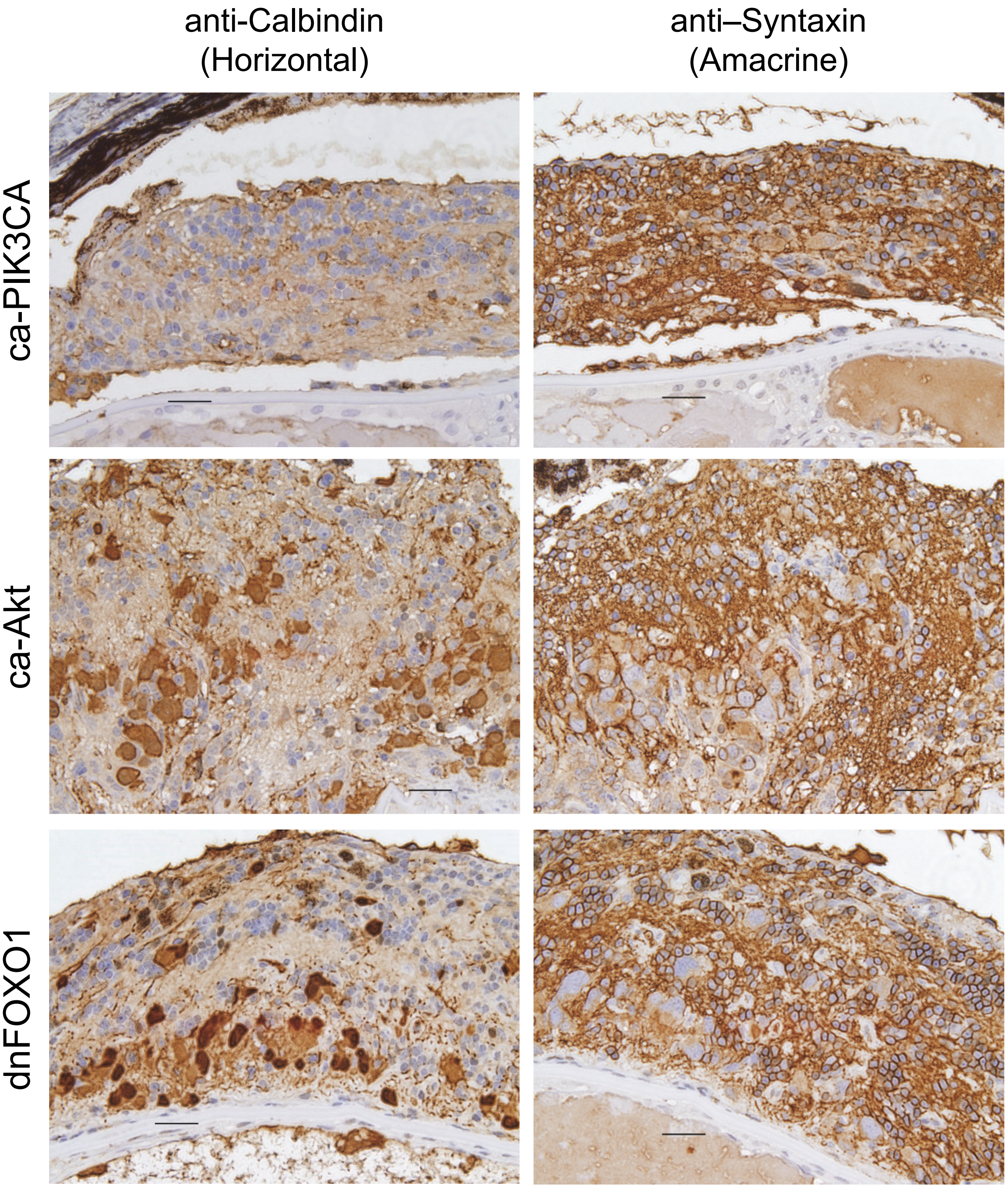

Figure S3, Related to Figures 2. ca-PI3KCA, ca-Akt, and dnFOXO1 induced tumors express horizontal and amacrine cell markers. Retinal tumors derived from *in vivo* electroporations using ca-PIK3CA, ca-Akt, or dnFOXO1 plasmids were sectioned and analyzed for IHC with anti-calbindin or anti-syntaxin antisera. Scale bar, 20mm.

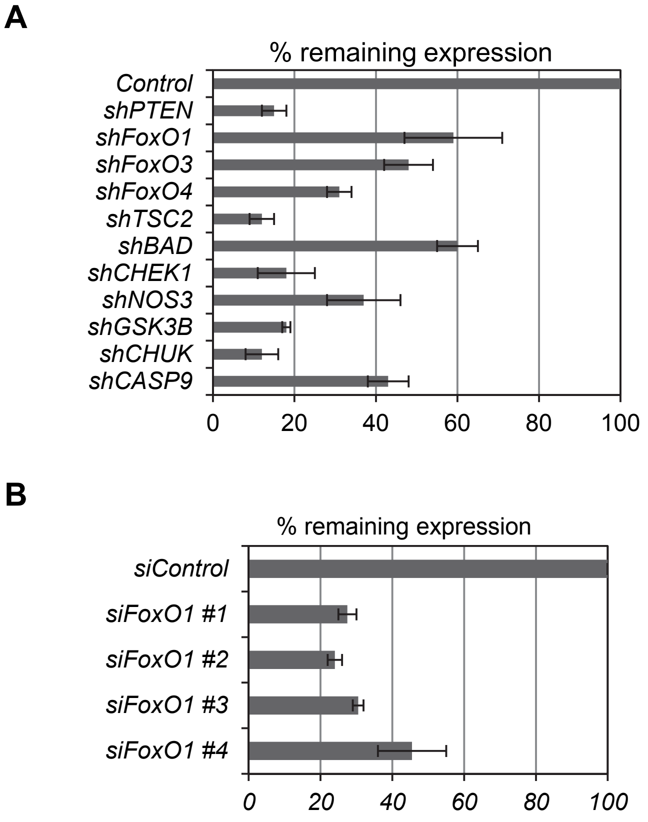

Figure S4, Related to Figure 4. Target gene knockdown levels by shRNA and siRNA. A. REF52 cells were stably integrated with shRNAs specific for the listed AKT phosphorylation targets and measured for target knockdown by qPCR. **B.** U2OS cells were transfected with control or four individual FoxO1 siRNAs and RNA isolated for qPCR detection of FoxO1 levels.

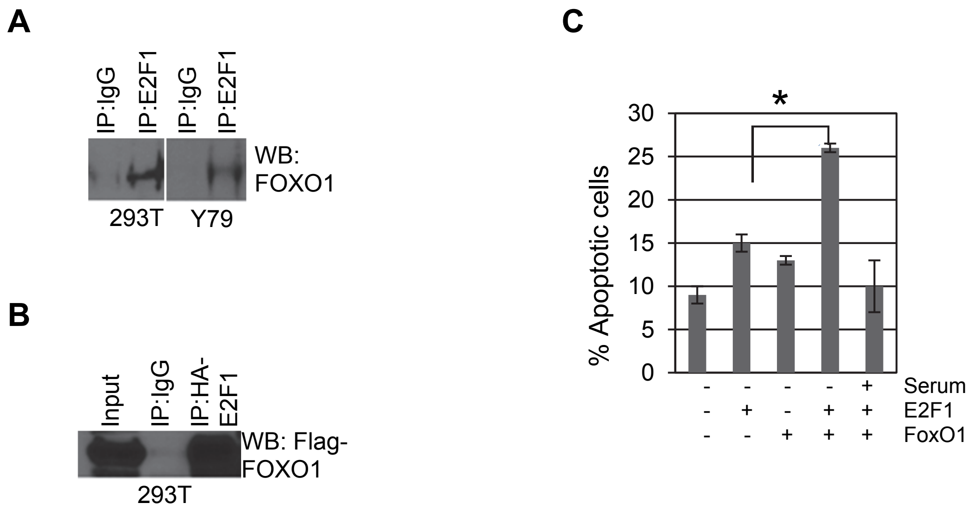

Figure S5, Related to Figure 4. E2F1 and FoxO1 physically associate and promote cell death. A. Endogenous co-IPs. 293T human embryonic kidney and Y79 human retinoblastoma cells were lysed, and the extracts were immuno-precipitated with anti-E2F1 antisera and immunoblotted for FoxO1. We conclude that endogenous E2F1 and FOXO1 physically associate in these cell lines. **B. Tagged co-IPs.** 293T cells were transfected with HA-E2F1 and Flag-FoxO1, lysed, immunoprecipitated with anti-HA and blotted with anti-FLAG. **C.** E2F1 and FOXO1 were introduced into 0.25% (-) or 10% (+) serum treated U2OS cells and apoptosis measured. Their co-expression caused cell death in >25% of serum-deprived cells, whereas E2F1 or FOXO1 alone caused around 13% of the cells to induce apoptosis. E2F1 and FOXO1 do not induce cell death in 10% serum. These data are consistent with a model in which E2F1/FoxO induce apoptosis specifically under growth factor deprived conditions.

A. shRNAs used in this study

	A	B	C	D
PTEN	TRCN0000002746	TRCN0000002747	TRCN0000028992	TRCN0000028993
FOXO1	V2LHS_169297	TRCN0000020707		
FOXO4	TRCN0000039721	TRCN0000071558		
TSC2	TRCN0000042723	TRCN0000042723		
BAD	TRCN0000009700			
Chek1	TRCN0000012651	TRCN0000012652		
NOS3	V2LMM_30746	V2LMM_217274	TRCN0000075867	
GSK3B	V2LMM_37597	TRCN0000000822	TRCN0000010551	TRCN0000012617
CHUK	TRCN0000000506	TRCN0000012349	TRCN0000012352	
Casp9	TRCN0000012249	TRCN0000012252		

B. Primers used in study

Gene	Sense Primer	Antisense Primer
TP73	GGCTGCGACGGCTGCAGAGC	GCTCAGCAGATTGAACTGGGCCATG
$p14^{ARF}$	CCCTCGTGCTGATGCTACTG	CATCATGACCTGGTCTTCTAGGAA
$p27^{Kip1}$	GGTTAGCGGAGCAATGCG	TCCACAGAACCGGCATTTG
FOXO1	TCGTCATAATCTGTCCCTACACA	CGGCTTCGGCTCTTAGCAAA
Bim	TGGCAAAGCAACCTTCTGATG	GCAGGCTGCAATTGTCTACCT
Apaf-1	AAGGTGGAGTACCACAGAGG	TCCATGTATGGTGACCCATCC
18S (control)	GTAACCCGTTGAACCCCATT	CCATCCAATCGGTAGTAGCG

Table S2. shRNAs (A) and primers (B) used in this study.

**Antibody**	**Cell type(s)**	**Species**	**IHC Dilution**	**Source**
Calbindin	Horizontal	Mouse monoclonal	1:500	Sigma (C9848)
Syntaxin (HPC-1)	Amacrine	Mouse monoclonal	1:8000	Sigma (S0664)
CHX10	Bipolar, RPCs	Sheep polyclonal	1:250	Exalpha Bio (X1180P)
PKCa	Bipolar	Mouse monoclonal	1:2000	Sigma (P5704)
Brn3b	Ganglion	Goat polyclonal	1:200	Santa Cruz (sc-6026)
CRALBP	Müller glia	Mouse monoclonal	1:500	Abcam (ab15051)
Rho4D2	Rod	Mouse monoclonal	1:800	Novus (NBP1-48334)
Cone Arrestin	Cone	Rabbit polyclonal	1:10,000	EMD Millipore (AB15282)

Table S2. Retinal Marker Antibodies used in this study.
